# Evolutionary origins of insulin resistance: a behavioral switch hypothesis

**DOI:** 10.1186/1471-2148-7-61

**Published:** 2007-04-17

**Authors:** Milind G Watve, Chittaranjan S Yajnik

**Affiliations:** 1 Anujeeva Biosciences Pvt. Ltd., 10, Pranav Soc. 1000/6-c Navi peth, Pune 411030, India; 2 Department of Microbiology, Abasaheb Garware College, Pune 411004, India; 3 Director, Kamalnayan Bajaj Diabetology Research Centre, King Edward Memorial Hospital, Pune 411011, India

## Abstract

**Background:**

Insulin resistance, which can lead to a number of diseases including type 2 diabetes and coronary heart disease, is believed to have evolved as an adaptation to periodic starvation. The "thrifty gene" and "thrifty phenotype" hypotheses constitute the dominant paradigm for over four decades. With an increasing understanding of the diverse effects of impairment of the insulin signaling pathway, the existing hypotheses are proving inadequate.

**Presentation of the hypothesis:**

We propose a hypothesis that insulin resistance is a socio-ecological adaptation that mediates two phenotypic transitions, (i) a transition in reproductive strategy from "r" (large number of offspring with little investment in each) to "K" (smaller number of offspring with more investment in each) and (ii) a transition from "stronger to smarter" or "soldier to diplomat" i.e. from relatively more muscle dependent to brain dependent lifestyle. A common switch could have evolved for the two transitions since the appropriate environmental conditions for the two transitions are highly overlapping and interacting.

**Testing the hypothesis:**

Gestational insulin resistance diverts more energy through the placenta, resulting in increased investment per offspring. On the other hand, insulin resistance is associated with reduced ovulation. The insulin signaling pathway is also related to longevity. Insulin resistance diverts more nutrients to the brain as compared to muscle. Also, hyperinsulinemia has direct positive effects on cognitive functions of the brain. The hypothesis gets support from known patterns in human clinical data and recent research on the molecular interactions in the insulin signaling pathway. Further we state many predictions of the hypothesis that can be tested experimentally or epidemiologically.

**Implications of the hypothesis:**

The hypothesis can bring about a significant change in the line of treatment as well as public health policies for the control of metabolic syndrome.

## Background

The Insulin Resistance Syndrome (IRS), also called metabolic syndrome or syndrome X, consists of a cluster of conditions including hyperinsulinemia, insulin resistance, impaired glucose tolerance, type 2 diabetes, hypertension, atherosclerotic vascular disease and coronary artery disease [1,2]. Insulin resistance is a condition in which tissues exhibit reduced response to insulin. This is accompanied by increased insulin synthesis resulting in hyperinsulinemia which is thought to be a compensatory response to reduced insulin sensitivity. A preliminary diagnostic criterion for insulin resistance is high levels of plasma insulin in relation to glucose levels. It is believed that the ability of the pancreatic beta cells to meet the demand posed by progressive insulin resistance ultimately reaches its limits. The failure of beta cells to secrete sufficient quantities of insulin leads to type 2 diabetes [3]. Obesity is positively correlated with insulin resistance and IRS is one of the major causes of mortality in the modern world.

Over four decades ago, James Neel speculated that the tendency to develop insulin resistance is unlikely to be a genetic disorder [4]. Instead, it must have evolved as an adaptive trait that later turned pathological due to changed life style and diet. He hypothesized that a "thrifty" genotype that helped survival in primitive life characterized by periods of "feast and famine" has now turned detrimental in the modern urban lifestyle and diet. Neel's hypothesis and its modified versions have dominated thought about the etiology of recent epidemic of diabetes.

### Inadequacies of the thriftiness hypotheses

The thriftiness family of hypotheses believes that the thrifty tendency minimizes energy loss and facilitates efficient energy storage in the form of fat during the days of "feast" and helps survival during a subsequent "famine". However, under conditions of stable and ample food supply that is characteristic of modern urban life, this tendency leads to obesity and related disorders. A presumption in Neel's original hypothesis was that a "quick insulin trigger" after meals helps conserve energy [4]. For this hypothesis to work it is necessary that the tissues respond to the increased levels of insulin by increased uptake of glucose. However, it was revealed a few years later that unlike Neel's presumption, loss of insulin sensitivity of tissues or insulin resistance is central to the syndrome [1,2] and therefore Neel's "quick insulin trigger" does not adequately explain the mechanism of energy conservation. Despite this failure, Neel's original thought of "thriftiness" remained dominant albeit with a modified meaning. In the new interpretation insulin resistance was thought to take the role of quick insulin trigger and serve the same purpose.

Later the genetic nature of the thrifty tendency was questioned [5] and a "thrifty phenotype" hypothesis stemmed from the finding that low birth weights predict the development of diabetes later in life [6]. A strong association between low birth weight and increased risk of diabetes and associated disorders has been found across a large number of geographical areas and ethnic groups throughout the globe [7-13]. The thrifty phenotype hypothesis differs from the thrifty gene hypothesis in that it does not assume the predisposing factors to be genetic but programmed by the intrauterine environment. According to the hypothesis, intrauterine undernourished environment induces thrifty mechanisms because it predicts a future of starvation. The thriftiness family of hypotheses has dominated evolutionary thought for over four decades. However, the validity of these hypotheses was never rigorously tested, mainly because researchers in this field have been primarily interested in medicine and epidemiology more than the basic evolutionary biology. When viewed critically a number of inherent weaknesses in these hypotheses become apparent.

1. The association between obesity and insulin resistance is very strong and universal, but the direction of the arrow of causation has been a source of ambiguity. The thrifty hypotheses presume that the function of the thrifty gene is to promote fat accumulation [14]. Neel (1962) wrote, "... the overweight individual of 40 or 50 with mild diabetes is not so much diabetic because he is obese, as he is obese because he is of a particular (diabetic) genotype" [4]. The key question is: can we equate the diabetic tendency to thirftiness? Is insulin resistance the thrifty gene (or phenotype)? Evidence for this is contradictory and inconsistent. In Pima Indians the most insulin sensitive individuals had a greater tendency towards weight gain than the most insulin resistant ones [15] and low insulin levels were better predictors of weight gain [16]. Hyperinsulinemia accompanying insulin resistance can possibly arrest weight gain rather than promote it by increased sympathetic nervous system activity and postprandial thermogenesis [17]. Mice with fat specific disruption of the insulin receptor have reduced obesity [18]. Insulin resistance induced by Klotho gene over-expression is accompanied by reduced adiposity [19]. Contradicting this, other mechanisms have been suggested by which insulin resistance may facilitate fat storage. Insulin exerts an antilipolytic effect in adipocytes and presumably this effect is relatively preserved in otherwise insulin resistant state [20]. Loss of neuronal insulin-leptin signaling can stimulate weight gain, but this is mainly through hyperphagia rather than metabolic frugality [3]. The basic tenet of the thriftiness hypothesis that insulin resistance contributes to a thrifty metabolism leading to energy storage is not unanimously supported. A number of molecular mechanisms are now known by which adipocytes actively modulate insulin resistance [20-23]. Surgical removal of fat reverses insulin resistance rapidly [24]. Weight loss can prevent progression from impaired glucose tolerance to type 2 diabetes [25]. The prevalence of obesity and IRS have rapidly increased and it is more logical to assume that increased obesity owing to increased energy intake [26] has resulted into increase in insulin resistance. On the other hand it would be absurd to assume that genes responsible for insulin resistance have increased owing to some unknown reason leading to increase in obesity. Thus although there might be a positive feedback cycle between adiposity and insulin resistance, the arrow of causation seems to be more strongly from adipocity to insulin resistance.

2. Implicit in the word thrifty is the concept of increased metabolic efficiency which would facilitate energy saving and storage. The prediction of the thrifty phenotype hypothesis is that muscle tissues of people with low birth weight should have a lower resting rate of metabolism. Evidence for this is contradictory. Lower resting metabolic rate was found to be associated with low birth rate in a small sample [27], whereas measurements on a larger sample size showed the opposite trend [28]. Therefore the assumption that low birth weight gives rise to thriftiness is not unanimously empirically supported.

3. The colder climates with harsh winters must have provided an ideal "feast and famine" situation of the thrifty gene hypothesis throughout major part of human evolution. In such climates hunting-gathering or agriculture is possible only for a few months of the year and for the rest survival depends upon stored food. Therefore insulin resistance should be more common in races that evolved in highly seasonal environments. Although it is difficult to correct for dietary and cultural differences in a cross-cultural study, the gross pattern emerging from the existing epidemiological data do not support this prediction of the thrifty gene hypotheses. Upon urbanization, the incidence of IRS in ethnic groups of tropical origins is the highest, and gradually reduces in people from harsh winter habitats. The European whites have a substantially lower incidence [16,29] and Iceland has a particularly low and stable incidence [30]. Eskimos have low incidence [31]. Urbanized Asians have a very high prevalence [32] but within urbanized Asians, Japanese have lower prevalence [33]. Within the Indian subcontinent, the migrant Tibetans that came from a harsh cold climate into tropical peninsula have a lower tendency for central obesity than the peninsular Indians [34]. The urbanized people of Kashmir-Himalayas also have a lower prevalence of diabetes than the rest of the urban Indians [35]. It seems a general norm that people adapted to harsh winter environments for several generations have lower tendencies to develop insulin resistance. This is contrary to the expectation of the thrifty gene hypothesis.

4. O'Rahilly and Farooqi [36] recently reviewed the mode of action of all known genes predisposing to obesity and concluded that human obesity was less metabolic and more neuro-behavioral disorder. Genetic and molecular differences between obese and non-obese rats also revealed that the major differences are neuro-behavioral rather than metabolic [37]. The relative contributions of metabolic thriftiness and neuro-behavioral factors to the current obesity epidemic are not yet critically evaluated.

5. In recent years our understanding of the molecular basis of insulin action and the insulin signaling pathway has expanded dramatically. We now know that insulin signaling is not only involved in energy homeostasis but also in longevity, reproduction, immunity as well as brain development and function. Insulin resistance is shown to alter reproduction, immunity and brain function. The thriftiness family of hypotheses has not provided adequate adaptive explanations for these aspects of insulin signaling and insulin resistance. For example castration in male rats induces insulin resistance and obesity [38,39] and the existing evolutionary paradigm offers no adaptive explanation for this. Therefore the thrifty gene and thrifty phenotype hypotheses are certainly inadequate if not wrong.

## Presentation of the hypothesis

Apart from thriftiness by fat storage and utilization, other mechanisms by which insulin resistance can aid survival under specific conditions have been suggested. Different tissues differ in their dependence on insulin for glucose uptake, skeletal muscles, liver, fat cells and abdominal viscera being among the most insulin dependent and brain, red blood cells and placenta being among the most insulin independent ones. By varying the levels of insulin and the degree of insulin sensitivity of tissues the energy budget allocation to different organs can be finely manipulated. Insulin metabolism is likely to have evolved primarily as a mechanism of differential energy allocation to different tissues. High insulin resistance or a low level of insulin would divert more energy to the insulin independent tissues and less to insulin dependent tissues [16]. Reaven [1] suggested that under conditions of starvation, insulin resistance makes more glucose available to the brain.

With the presumption of a role of insulin metabolism in differential allocation of energy, we develop a hypothesis that insulin resistance evolved as a socio-ecological and socio-nutritional adaptation rather than thriftiness. We agree with the component of Neel's hypothesis that traits that were adaptive in primitive life are turning detrimental today, but differ in explaining what was adaptive and why it is detrimental today. We propose that insulin resistance can alter the behavioral and reproductive strategies of an individual. This is achieved on the one hand by modifying the energy budget allocation to tissues and on the other by the direct action of insulin on different tissues. We propose that insulin resistance can bring about two phenotypic transitions that we call "r to K strategist" and "stronger to smarter" respectively. This hypothesis explains almost all the physiological findings and epidemiological patterns that are not explained by the thriftiness hypotheses.

### The r to K transition in reproductive strategies

Population biology and evolutionary theory has extensively used the logistic model of population growth and r and K are the two parameters of the model. The intrinsic growth rate of a species is denoted by r; and K stands for the carrying capacity of the environment for the species [40,41]. The r and K strategies are classically described as two alternative ways of gaining evolutionary fitness. An r strategist is one that reproduces faster by producing a large number of offspring, investing little in each. A K strategist on the other hand produces fewer offspring but invests more in each [40-43]. Initially the concept was used across species but it was shown that this plurality in reproductive strategies can exist within a species as well [42,44]. Natural selection would favor an r strategist when there are ample opportunities for population expansion and intraspecific competition is minimum. The K strategy will be favored when the population is close to the carrying capacity of the environment resulting into fewer opportunities for survival [42,43]. Evolving in an environment that fluctuates in its selective forces, it would be highly adaptive to be able to switch between alternative reproductive strategies by a physiological or behavioral switch [44]. Insulin signaling can provide such a switch. Placenta is an insulin independent organ and therefore gestational insulin resistance is expected to divert more nutrients through the placenta. Maternal insulin resistance is known to result into larger babies [4,45]. The larger birth weights do not simply reflect weight gain by mothers. Catalano et al [46] demonstrated that in normal mothers the fetal weight gain was positively correlated with maternal weight gain, but in diabetic mothers increase in fetal weight was independent of maternal weight gain. We hypothesize therefore that insulin resistance is the mechanism by which the investment in a fetus is increased. On the other hand insulin resistance is also known to reduce ovulation [47-50]. Insulin resistance is a strong contributor to polycystic ovarian syndrome (PCOS) and insulin-sensitizing drugs are the most effective agents to reverse anovulation due to PCOS [51-53]. Insulin signaling is involved in reproduction by direct endocrine as well as neuronal action [52]. Although some important links in the mechanism are not completely understood, the duel action of insulin resistance i. e. reducing fertility on the one hand and increasing fetal growth on the other is well supported.

The r and K selection strategies in reproduction are also accompanied by differences in lifespan. The r strategists are typically short lived and K strategists long lived. In mammals body size and longevity correlate positively with each other and negatively with the rate of reproduction [54,55]. It makes sense therefore that insulin signaling is also connected to longevity. In *Caenorhabditis elegans *mutations of genes involved in insulin/IGF-1 signal transduction lead to extended life span [18,56]. Mutations in genes homologous to the mammalian insulin/IGF-1 receptor signaling pathway, insulin receptor substrate homologue *chico *or insulin like receptor InR gene extend longevity in *Drosophila *[18]. In mammals and particularly humans insulin is certainly connected to longevity, but in a rather contradictory way. On the one hand insulin resistance is associated with a series of fatal disorders and centenarians are known to have high insulin sensitivity [18], but on the other, the Klotho gene whose overexpression is known to increase lifespan has been shown to act by increasing insulin resistance [19]. FIRKO mice with fat cell specific insulin resistance have a prolonged life span [57]. Resveratrol, a plant derived compound that is known to prolong lifespan in yeast, *C. elegans *and *Drosophila*, and now shown to be active in mammals as well, also acts by inhibition of insulin signaling pathway [58]. On the contrary life extension in dwarf mice is associated with reduced secretion of insulin and increased hepatic sensitivity to insulin [59]. The key differences in the contradictory results might lie in differential tissue specific resistance to insulin. Currently the association of insulin resistance with longevity in humans remains ambiguous. However there is strong evidence that insulin signaling and insulin resistance is certainly involved in determining longevity in widely differing animals. The strong influence of insulin resistance in reproductive and life history strategies therefore must have played a major role in the evolution of insulin resistance.

### The "stronger to smarter" or "soldier to diplomat" transition

We suggest that insulin resistance mediates a shift from muscle dependent to brain dependent strategies of making a livelihood. This is achieved partly by changing the relative energy allocation to muscle versus brain and partly by insulin signaling in the nervous tissue. Insulin resistance is characterized by reduced glucose uptake by muscle and viscera resulting into high plasma glucose levels and since the uptake of glucose by brain cells is almost independent on insulin levels in the physiological range [60,61], more glucose is available for the brain. Studies are needed to test whether this extra energy made available by insulin resistance positively affects the brain function, but an alternative mechanism by which brain function is enhanced has substantial experimental evidence. Insulin resistance is also characterized by hyperinsulinemia. Insulin receptors are widely distributed in specific brain areas including choroids plexus, olfactory bulb, pyriform cortex, amygdaloid nucleus, hippocampus, hypothalamic nucleus and cerebellar cortex. The insulin receptor in the central nervous system differs both in structure and function from the peripheral tissue receptor [62]. The brain also synthesizes some amount of insulin, but plasma insulin levels do affect brain functions substantially. Insulin and insulin receptor in the CNS is associated with neuronal development [63] as well as a number of cognitive functions including spatial and verbal memory [62]. Streptozotocin, which inhibits insulin receptor function causes long term diminution in memory in rats on intracerebroventricular administration [64]. Insulin signaling plays a significant role in cognitive functions of the brain. Peripheral insulin resistance accompanied by high levels of insulin can therefore be said to be good for brain function. Two other molecules which, similar to insulin, were thought to be important in energy metabolism alone, and which play a significant role in IRS are now shown to enhance cognitive functions, namely leptin [65] and cholesterol [66].

Contrary to the positive effects of raised insulin levels on brain function, diabetic patients have increased risk of developing dementia and Alzheimer's disease (AD) [67-69]. However, these effects are observed only in old age subjects [70]. Furthermore, it is likely to be a result of local insulin deficiency rather than sustained high levels of insulin [71,72], since in AD patients the csf to plasma insulin ratio is particularly low and negatively related to the severity of AD [73]. Elevated insulin is shown to enhance memory in AD patients [74] and intranasal insulin has been advocated as a treatment for AD [75,76]. It is possible that impairment of insulin receptors at the blood brain barrier [77] may be central to the adverse effects on the CNS.

In young and middle age, adverse effects of hyperinsulinemia or insulin resistance on brain function are rare [70]. On the contrary, there are positive short term effects. If the negative effects are at an age well past the reproductive age, natural selection is unlikely to be affected by them. On the other hand the positive effects at an early age would be strongly selected for. Therefore it is a logical proposition that hyperinsulinemia would have selective advantages for a brain dependent lifestyle.

Further, high levels of insulin are shown to slow down pre-attentive responses simultaneously enhancing attentive responses [78]. Research in this direction is limited, but it raises a possibility that insulin suppresses fast motor reflexes and enhances relatively slower functions such as memory and thinking. If this generalization is true it is in support of our hypothesis. Fast reflexes are typically required in soldier life and thinking in diplomat life. The raised plasma insulin levels in insulin resistance may bring about this subtle transition as well.

It is not surprising that reduced physical activity tends to induce insulin resistance or type 2 diabetes and that muscle exercise can partly revert insulin resistance [11,79,80]. It is logical to allocate a reduced budget to an organ that is less active and increase the budget allocation when its consumption increases. However, there is an additional possible mechanism through which muscle insulin resistance may be adaptive. Water maze trained mice were shown to up-regulate insulin signaling in the hippocampus [81]. It is possible therefore that when intense brain activities are consistently required the body may respond by making more insulin as well as more insulin receptors in the CNS. However, since higher plasma insulin levels result in hypoglycemia and hypoglycemia is detrimental to brain function, peripheral insulin resistance develops to maintain higher plasma glucose. Hyperinsulinemia is known to down-regulate insulin receptor activity in peripheral tissues but not in the nervous tissue [77] and this differential loss of sensitivity presumably enhances brain activity at the cost of muscle activity. According to the classical belief, insulin resistance is a primary response and hyperinsulinemia develops as a compensatory response. We are tempted to suggest that hyperinsulinemia to enhance brain function may develop primarily in people with a sedentary and brain centered life style. However hyperinsulinemia may result into hypoglycemia which is detrimental to brain function [82,83] and to prevent this, peripheral insulin resistance develops as a compensatory adaptation.

The soldier to diplomat transition also explains the association of low birth weight with insulin resistance in later life. Muscle cells do not multiply in adult life and nor do nerve cells. If nutrient limitation affects intrauterine development, brain development is the least affected among all the organs [84,85]. As a result, if the intrauterine growth is slow or impaired, muscle weight is poor but the brain is relatively well developed. For such an individual, facing a competitive environment in adult life, trying to be smarter is a better bet than trying to be stronger. The muscle mass being poor, competing with others in muscle activity is unlikely to succeed. On the other hand brain development being normal, such a person is more likely to compete successfully in brain related activities. An individual born small for gestational age therefore should take to a diplomat life rather than a soldier life and insulin resistance is adaptive for such an individual.

If there are no developmental constraints during intrauterine or early infant development there need not be a trade-off between body physique and brain development. A positive association between IQ and physique has been shown in twin studies and the causes claimed to be genetic [86], but intrauterine factors could play a substantial role. However, with developmental constraints such as intrauterine malnutrition, trade offs are unavoidable and a negative association between physique and IQ is more likely to develop. Developmental constraints would affect both brain and physique, but not equally. The brain sparing effect and priority to brain over muscle becomes inevitable in these circumstances and we suggest that insulin resistance is a mechanism by which this shift in balance during development is maintained in adult life as well. Adopting a brain centered life need not necessarily mean having a higher IQ just as people with more muscle dependent life style such as hunters or laborers are not necessarily the most highly muscular persons. For development compromised individuals, since brain development is the least affected, a brain centered life is the best out of the bad options.

In primate societies individuals start gaining a position in the social hierarchy among their age group from a very early age [87-90]. Therefore we expect the childhood growth rate to be a surrogate of the developing social rank, and a significant positive correlation between growth rate and insulin resistance is not surprising [11]. Individuals born small but growing fast have a triple reason for developing insulin resistance. On the one hand, owing to smaller muscle mass they are unlikely to succeed as soldiers and have to adapt a diplomat way of living. On the other hand, the rapidly gained better social ranking would drive them further towards a K and smarter phenotype for reasons described below. Small birth weight might also be taken as a cue to overpopulation and competition for food, which can be another trigger for adopting K and smarter strategies. If the three triggers act independently or additively, there would be a high risk for "born small grown big" individuals [11].

In an apparently contradictory way predisposing factors to insulin resistance include both overeating and malnutrition. It is possible to explain both with a coherent logic. When foraging success is a limiting factor for the fitness of an individual it is necessary to invest in muscle to enhance foraging activity. When food is ample, fitness would depend more on social interactions than on foraging for any social species. Disinvestment from muscle and increased investment in brain towards gaining higher social manipulation skills would be adaptive under these conditions. On the other hand in extreme malnutrition and fetal malnutrition in particular, the available nutrients should be preferably diverted to the brain as a minimum survival need. The 'selfish' brain itself actively manipulates resource allocation under certain conditions [91]. The mechanisms of preferential allocation to the brain include insulin resistance. Thus insulin resistance should be adaptive under both extremes of nourishment.

#### Why a common switch for two phenotypic transitions?

**Figure 1:**
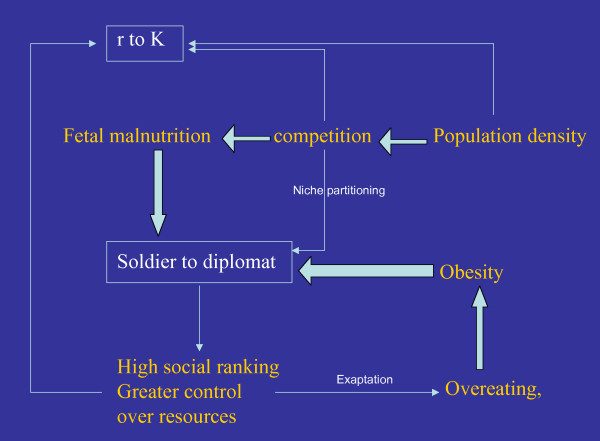
**Causal relationships between environmental factors and the two transitions**. Thick arrows indicate causations for which there is adequate evidence currently. Thin arrows are the causations suggested by our hypothesis that need to be tested.

Although being smarter and being K selected are two independent strategies, there is a marked similarity in the socio-ecological conditions under which a smarter phenotype would get selected and those under which a K strategist would get selected. The two transitions are also likely to interact with each other as schematically shown in figure 1. Therefore a common switch for both could have evolved. A high population density is a natural condition for changing reproductive strategies. High population density also leads to more intense competition and niche separation is one of the effective ways to handle competition. The soldier-diplomat dichotomy can be viewed as a way of niche separation. Competition also increases the variance in individual successes, leading to a greater hierarchy or social divide. This would leave some section of the society malnourished and some with a greater control over resources. Chronic malnutrition is also a predisposing factor for insulin resistance, particularly fetal malnutrition for reasons explained earlier. In a saturating environment all individuals would not be equally quick to adopt a K strategy. A policy of cutting down the number of offspring can be successful only if the probability of offspring survival is sufficiently high. In social organisms the high-ranking individuals are relatively less risk prone and have a greater control over resources improving their chances of survival in crisis. Therefore we expect the high social rank individuals to preferentially adopt a K strategy. The high-ranking individuals are also in a better position to manipulate other individuals in the social group and so can be more successful as diplomats. Successful diplomats in turn have greater control over resources and should be in a better position to ensure offspring survival and so preferentially adopt a K strategy.

An interesting possibility, although quite speculative at this stage is that of exaptational overeating and obesity. Money is a very recent phenomenon in the evolutionary history of humans and therefore separate brain centers to handle money related emotions and information processing are unlikely to have evolved. The brain areas involved in handling food related emotions and information were presumably exapted to handle money. Therefore there could be a cross talk between the neural mechanisms of handling money and food. It's known that the region of the orbitofrontal cortex involved in processing food rewards is also involved in processing money rewards [92-94]. Briers *et al *showed that under experimentally manipulated situations hunger affects money related decisions and the desire for money increases hunger [95]. It is also possible that the desire to accumulate wealth results into a tendency to store fat. This interesting possibility generates many key questions and needs a deeper probe. Currently studies in this direction are limited and we do not intend to lay more emphasis on it other than stating that this is another possible way by which socioeconomic and neuro-physiological processes could interact.

Thus the triggers for the two transitions are not nutritional alone but social, behavioral, economic and ecological. The insulin resistance syndrome has traditionally been viewed as a nutritional disorder. We would like to emphasize that apart from diet, other factors can also be an important consideration in management and control of metabolic disorders. The predisposing factors for IRS are not only sedentary life and rich diet but also local population density, competitive social environment, strong economic motives, social divide, social manipulation skills and the tendency to avoid physical aggression.

#### Why an association between obesity and insulin resistance?

Adipocytes have a crucial role in IRS. Obesity is a prime risk factor for diabetes and related disorders. There are a number of possible reasons compatible to our hypothesis for an association between adiposity and insulin resistance.

1. The brain consists of 60–70% fat by dry weight and lipid metabolism is shown to affect brain development, intelligence, and behavior [96-100]. Insulin signaling is suspected to play a role in myelination of nerve fibers [77] and myelin sheaths are predominantly composed of fat. Fat storage can be viewed as a raw material buffer in brain development and function.

2. Fat accumulation can be a cue for high social ranking in traditional human societies and high social ranking is a prerequisite for transition to K selection.

3. Adiposity can work as a social signal. The theory of honest signaling or the "handicap principle" emphasizes the importance of giving honest signals of one's strategies and intensions and that signals with high cost are more honest [101]. A smarter individual would avoid physical aggression and gain fitness by manipulating other individuals. In order to avoid physical fights an honest signal has to be passed on to other individuals. A change in body proportions (central obesity) can be a fast and efficient signal since peripheral fat is more difficult to differentiate from muscularity than abdominal fat. It makes sense therefore that central obesity is more strongly correlated with insulin resistance than peripheral fat (2,7). Fat storage has a high energy cost making it a more honest signal.

4. Obesity adversely affects agility which is an important requirement of soldier life. An obese individual is unlikely to be a successful soldier and therefore would find a diplomat transition more adaptive.

### The origins of pathology

We have argued throughout that insulin resistance is an adaptive response. Furthermore at least under certain conditions insulin resistance can actually increase the life span [19]. On this background, we need to answer the question, why a tendency that was adaptive, has turned into a pandemic disorder today. We feel that the pathological effects are caused by supernormal stimuli. All the stimuli for development of either or both the phenotypic transitions such as population density, social competitiveness, economic motives, social inequality, sedentary life and rich diet have reached unprecedented levels today to which the body gives an exaggerated response leading to pathological effects.

Of particular interest are the immunological changes accompanying obesity and insulin resistance. We suggest that the distribution of the immune system within the body changes with the behavioral strategies of an individual. It has been suggested that the apparent immunosupression accompanying high testosterone levels is not a "suppression" of the entire immune system but a redistribution of it [102]. Since high testosterone levels are associated with male aggression, components of the immune system migrate to the subcutaneous tissues anticipating fights and injuries. In a soldier to diplomat transition we expect a redistribution response in the reverse direction in which components of the immune system are withdrawn from the subcutaneous tissues as physical fights are less likely. This can result into increased sensitivity to inflammation in the central tissues and a delay in cutaneous wound healing. An exaggerated redistribution response in this direction may make the central tissues more inflammation prone. Inflammatory changes are increasingly being recognized as a major contributor to the comorbidities of the metabolic syndrome. Anti-inflammatory drugs can reverse at least some of the components of the insulin resistance syndrome [103,104]. Atherosclerosis involves inflammation of the vascular epithelium [105-108]. Adipocytes produce many signal molecules including IL-6, TNF alpha and C reactive protein that enhance inflammatory response. Concentrations of these immune markers are positively correlated to measures of total and particularly central obesity [109]. Chronic inflammation is suspected to be the main mechanism of adiposity induced insulin resistance, fatty liver disease, airway inflammation and atherosclerosis [110-113].

There is evidence that loss of pancreatic beta cell function that underlies clinical diabetes is also induced by inflammation. Glitazones that have been used in the treatment of type 2 diabetes act as PPAR-gamma agonists. On binding with them PPAR-gamma forms a heterodimer that can interfere with proinflammatory pathways [114]. The beneficial effects of glitazones are likely to be via reduction in inflammation of the pancreas itself. The clinical onset of type 2 diabetes therefore may be a result of immune redistribution accompanying the soldier to diplomat transition. It is possible that some of the inflammatory reactions are actually triggered by subclinical infections. Mild infections can be the precipitating cause of morbid disorders but the root cause is likely to be the exaggerated immune redistribution response of the body.

The immune redistribution hypothesis is also supported by the negative association between testosterone levels and insulin resistance in males [38,39,115-122]. Rise in testosterone levels may reverse insulin resistance [118,120]. While testosterone is suggested to drive the immune system subcutaneous, insulin resistance according to our hypothesis, does the reverse.

It is very likely that primary and limited insulin resistance in which loss of peripheral sensitivity and raised insulin levels compensate each other can be good for health and longevity. Klotho gene induced insulin resistance is shown to increase longevity [19,123] but lipo-apoptosis, inflammatory changes and beta cell defects are the confounding factors leading to the cluster of diseases. It is likely therefore that controlling obesity and immunological changes would be a more effective therapeutic strategy than trying to reduce insulin resistance. A serious thought and thorough examination of this proposition is needed since, if proved to be correct, it can change the line of treatment of metabolic disorders substantially.

### Social hierarchy and insulin resistance

There is a positive correlation between obesity and insulin resistance in all the ethnic groups examined [2,7,29,80,124], and central obesity is even better correlated [7,124]. Obesity alone, however, does not explain the higher prevalence in higher income groups in tropical countries. In Indian studies the positive association of type 2 diabetes with income remained significant even after accounting for age and obesity [125,126], suggesting that social hierarchy may play a role independent of the nutritional status.

In an interesting experiment a Wistar rat colony exposed to chronic malnutrition was compared with a control colony [127]. The control colony was fed *ad libitum *and the malnourished colony was fed a limited diet only once a day. In effect the malnourished animals ate approximately 50 % in amount as compared to the control. The control colony diet was also higher in protein and fat. The malnourished animals showed exceedingly high levels of insulin than the control ones. In the malnourished individuals, the mean body weight was reduced but the brain weight was preserved. Remarkably, the malnourished animals accumulated disproportionately more fat than the control ones. Across the two groups there was a negative association between body weight and hyperinsulinaemia, but within the malnourished group there was a positive correlation. Among the malnourished group, animals with higher BMI were severely insulin resistant although their BMI were substantially lower than the control group with normal diet and whose insulin and glucose levels were normal. This suggests that relative obesity rather than absolute one played a major role. These apparently contradictory effects of body weight on insulin levels can best be explained by the K strategy hypothesis. Malnutrition is a cue to a saturating population, which is the appropriate time to adopt a K strategy. However within the malnourished, the heavier and so presumably high social ranking individuals should preferentially take to this strategy. The incidence of diabetes today is rapidly increasing in the third world countries and the affluent in these countries are at maximum risk [80,128-130], a picture that matches well with the rodent experiment [127].

There are other reasons to believe that, relative, rather than absolute, nutritional status is more important. Food rationing which reduces nutritional variance has resulted in a dramatic reduction in diabetes on a number of occasions [131]. In a number of studies, the prevalence of type 2 diabetes in urban areas was significantly more than rural ones [80,128-130]. In all these studies, obesity was more prevalent in the urban communities. However the regression coefficients of insulin resistance parameters with obesity parameters were higher in rural areas [129,130] although the intercept was substantially smaller. It is possible that the lower intercept was due to low population densities and more physical work in the rural areas. But the higher slope suggests that although rural communities were much less obese as compared to their urban counterparts, the relatively more obese individuals within the rural community were more prone to the disorder [11]. This suggests that relative, rather than absolute obesity matters. The thriftiness hypotheses are unable to explain the importance of relative obesity.

Contradicting this picture is the reversal of socioeconomic correlates of metabolic disorders in developed countries. Contrary to tropical Asia the correlation between socioeconomic status and metabolic disorders is negative in many developed countries [132-136]. We need a careful look at these data to see whether the trend remains after correcting for race, diet, smoking and other risk factors. Alley et al [137] show that the proportion of whites and non-whites was substantially different across socioeconomic groups. Racial differences may partly explain the negative socioeconomic trend. Contrary to the expectation of the feast and famine hypothesis ethnic groups of tropical origin appear to have a greater tendency to develop insulin resistance as compared to people from harsh winter areas [30-36]. In harsh winter habitats, hunting-gathering or agriculture was possible only for a few months of the year and during winter people were forced to take an indoor sedentary life and survive on stored food. Sedentary life is a known predisposing factor for metabolic disorders. We suggest that the forced seasonal sedentary life for several generations could have selected against the genes predisposing for metabolic syndrome in response to sedentary life. Therefore the obesity threshold for IRS in these people is higher than the tropical people who could remain active hunter-gatherers throughout the year and for whom sedentary life is only a modern phenomenon. Studies in which racial factors are adequately incorporated show a much weaker negative socioeconomic trend [138-140]. It is likely that since in the developed world even the relatively deprived class is not malnourished, and owing to mechanization most of the labor class also does little physical work, differences in nutrition and exercise are not similar to the less developed societies. Therefore other factors such as crowded living conditions may become important. Traditionally high fat diet is associated with high social ranking. However, in these societies the lower classes consume more fat and that could be the major confounding factor.

## Testing the hypothesis

Apart from being able to explain most of the known epidemiological patterns the hypothesis makes many more predictions that are potentially testable.

(i) Comparison across animal species: If insulin resistance is a social adaptation, more social species should show a greater tendency to develop insulin resistance. If, on the other hand, it is a thrifty adaptation, species evolved in highly seasonal environments, species that hibernate or migrate long distances and store substantial amounts of fat before hibernation or migration should show the highest tendency. Standardization of methods and definitions across species is difficult and there are no data for sound interspecies correlation currently. However, in the long run, a careful multispecies comparison can be enlightening in many ways.

ii) The fetal origins or thrifty phenotype hypothesis predicts that although a transition from traditional lifestyle to the urban western lifestyle is associated with a high prevalence of IRS, it will quickly reduce in subsequent generations as the birth weights improve [5]. Although the social adaptation hypothesis incorporates the effect of low birth weight, it predicts that improvement in birth weights alone will not wipe off the difference completely.

iii) According to the K selection hypothesis the incidence of insulin resistance should correlate positively with local population density after correcting for other risk factors. There is some evidence that obesity and type 2 diabetes are more prevalent in crowded cities but it hasn't been interpreted as an effect of crowding [141-143].

iv) Since the relative position in the social hierarchy is important it is possible that the prevalence of IRS could be low in nutritionally egalitarian societies. Societies with a greater rich-poor divide, particularly in the nutritional quality, should show a higher incidence.

v) The parental investment theory predicts certain gender differences in reproductive behavior [144]. The reproductive success of males can increase to a much greater extent with increase in inputs as compared to females. This is because high ranking males can gain access to more females and thus raise their total reproductive success. A corollary is that few other males will be left with little reproductive opportunities. The reproductive success of females on the other hand has limited variance. As a result investments in male and female offspring pay in different ways. If the increased fetal weight in gestational insulin resistance is an investment strategy, we expect a difference in male and female fetuses. Insulin resistant mothers should invest more in male than in female fetuses. As a result the relation between maternal insulin resistance and birth weight should be stronger and steeper for male than for female infants.

There are many sources of confusion and contradiction in our current understanding, the major one being about longevity versus fatality and adaptive versus pathological course. The key area on which more research inputs are needed is the differential effects of insulin resistance in different tissues. Tissue specific gene expression or knock out studies have already started contributing to our understanding. Apparently fat cell specific insulin resistance is "good" for health and longevity and hepatic and brain insulin resistance is "bad". The key question is that whether different triggers for insulin resistance affect different tissues in different ways. We need to know whether clinical progression of insulin resistance serially impairs the sensitivity in fat cells, muscle, liver, blood brain barrier and ultimately the brain. It is tempting to suggest that the former two may actually be good for health and the latter bad. Answering these questions could revolutionize the way of thinking with respect to the basic biology as well as clinical management.

## Implications of the hypothesis

The traditional view that insulin resistance evolved as a mechanism to combat periodic starvation is grossly inadequate and other interpretations of the phenomenon are possible. There is strong evidence that insulin resistance changes the reproductive and life history strategies on the one hand and life sustenance strategies on the other. Therefore insulin resistance is more likely to have evolved as a switch in reproductive and sustenance strategies than energy homeostasis alone. The pathological consequences of the syndrome are likely to be caused by the immune redistribution response rather than insulin resistance *per se*. If the hypothesis stands the tests, it can change the epidemiological thought and help us refine the line of action for control of the disorders associated with the Insulin Resistance Syndrome. Clearly substantial research inputs are needed to bring about a change in paradigm, but a possible change in the line of control and treatment policies can be perceived today. Since the insulin resistance syndrome is a major cause of morbidity and death throughout the world today, and since an evolutionary understanding has the potential to revolutionize the line of prevention and treatment of the disorder it would be a valuable example of contribution of evolutionary biology to medicine.

## Authors' contributions

CY recognized the inadequacies of the thriftiness hypotheses, initiated interaction between the authors and provided clinical and epidemiological data, MW formulated the hypothesis, analyzed literature and wrote the manuscript. Both the authors approved the final manuscript.
